# Metabarcoding and Metabolomics Reveal the Effect of the Invasive Alien Tree *Miconia calvescens* DC. on Soil Diversity on the Tropical Island of Mo’orea (French Polynesia)

**DOI:** 10.3390/microorganisms11040832

**Published:** 2023-03-24

**Authors:** Camille Clerissi, Slimane Chaïb, Delphine Raviglione, Benoit Espiau, Cédric Bertrand, Jean-Yves Meyer

**Affiliations:** 1 PSL Université Paris: EPHE-UPVD-CNRS, UAR 3278 CRIOBE, Université de Perpignan, 52 Avenue Paul Alduy, Cedex, 66860 Perpignan, France; 2 PSL Université Paris: EPHE-UPVD-CNRS, UAR 3278 CRIOBE, B.P. 1013, 98729 Papetoai, Mo’orea, France; 3 Délégation à la Recherche, B.P. 20981, 98713 Papeete, Tahiti, France

**Keywords:** bacteria, biological invasion, metabolites, metazoans, microeukaryotes

## Abstract

*Miconia calvescens* is a dominant invasive alien tree species that threatens several endemic plants in French Polynesia (South Pacific). While most analyses have been performed at the scale of plant communities, the effects on the rhizosphere have not been described so far. However, this compartment can be involved in plant fitness through inhibitory activities, nutritive exchanges, and communication with other organisms. In particular, it was not known whether *M. calvescens* forms specific associations with soil organisms or has a specific chemical composition of secondary metabolites. To tackle these issues, the rhizosphere of six plant species was sampled on the tropical island of Mo’orea in French Polynesia at both the seedling and tree stages. The diversity of soil organisms (bacteria, microeukaryotes, and metazoa) and of secondary metabolites was studied using high-throughput technologies (metabarcoding and metabolomics, respectively). We found that trees had higher effects on soil diversity than seedlings. Moreover, *M. calvescens* showed a specific association with microeukaryotes of the Cryptomycota family at the tree stage. This family was positively correlated with the terpenoids found in the soil. Many terpenoids were also found within the roots of *M. calvescens*, suggesting that these molecules were probably produced by the plant and favored the presence of Cryptomycota. Both terpenoids and Cryptomycota were thus specific chemicals and biomarkers of *M. calvescens*. Additional studies must be performed in the future to better understand if they contribute to the success of this invasive tree.

## 1. Introduction

Biological invasions are one of the major threats to both marine and terrestrial biodiversity at the global scale [1,2]. For example, invasive alien plants greatly alter the native plant diversity of a region by forming dense stands [3]. Island ecosystems in which geographic isolation is often associated with higher ecological specialization and endemism of resident organisms are considered more vulnerable to invasions [4,5,6]. French Polynesia in the South Pacific Ocean, which is made up of about 120 islands spread over an area equivalent to Europe, is included in one of the 35 global biodiversity hotspots [7]. However, it faces the invasion of a plant species that is among the hundred most harmful invasive species in the world [8], the small tree *Miconia calvescens* (Melastomataceae), which is native to the tropical rainforests of Central and South America. This species has colonized thousands of hectares of tropical rainforests on the island of Tahiti and the neighboring island of Mo’orea, where it forms dense monospecific stands [9], and threatens several dozen endemic plants in French Polynesia [10].

Until now, the studies carried out in French Polynesia have described the effects of *M. calvescens* at the plant-community scale [9,11], but the dynamics at play at the rhizosphere level remain poorly explored. However, the rhizosphere is a fundamental compartment of plants because it is a key area of nutritive exchanges and communications with microorganisms (bacteria and microeukaryotes) as well as with meiofauna and other plants [12]. Interspecific communication (allelopathy) occurs mainly via secondary metabolites, and it can lead to positive or negative interactions between organisms. In general, these interactions structure plant and microbial communities [12], and they are the result of long co-evolutionary processes [13]. Nevertheless, few studies have aimed to describe how invasive plants fit into already-existing interaction networks. This could be performed, in particular, with the supply of new “weapons” (secondary or microbial metabolites), which would cause the destabilization of the rhizosphere [13] and would contribute to the construction of a new ecological niche [14]. For example, it has been proposed that the destabilization of soil microbial communities could favor the invasive success in Europe of the alien tree *Acacia dealbata* (Fabaceae) [15].

This study thus aimed to understand how the invasive alien tree species *M. calvescens* affects the rhizosphere composition in the tropical high volcanic islands of French Polynesia, where it was first introduced in 1937. High-throughput technologies (metabarcoding and metabolomics) were used to describe its influence on microbial and meiofauna assemblages and on the composition of secondary metabolites within the rhizosphere. In particular, we sampled the rhizosphere of seedlings and trees from *M. calvescens* and other native and introduced woody plant species to identify the specific effects of this dominant invasive plant.

We expected to (i) find a higher effect of trees on the soil compared to seedlings, (ii) find specific microbial and metabolite compositions associated with each plant species, and thus (iii) identify chemicals and biomarkers specific to *M. calvescens*. These markers might be useful to better understand the success of this invasive tree and to propose management tools for terrestrial ecosystems.

## 2. Materials and Methods

### 2.1. Sampling Site and Plant Species

**Figure 1:**
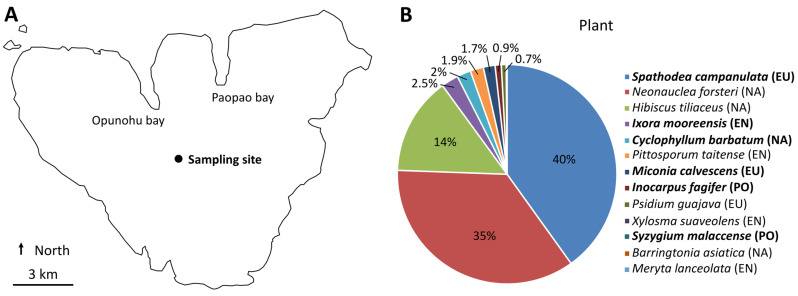
Sampling site in Mo’orea (French Polynesia). (**A**) Location of the sampling site. (**B**) Plant assemblage at the sampling site. The plant species of this study are in bold. EN: endemic; EU: European introduction; NA: native; PO: Polynesian introduction.

The sampling was performed in 2019 on the 7th of August on the island of Mo’orea (French Polynesia) at one site (GPS: 149°49′46.56″ W, 17°32′36.24″ S) located at ca. 250 m of elevation in the Opunohu valley (Figure 1A). Mo’orea is a 135 km^2^ high volcanic island that is between 1.15 and 2.45 Myrs old. It has a rugged topography with deep valleys, narrow ridges, and high peaks reaching 1207 m of elevation. It has a tropical oceanic climate with a mean annual temperature and rainfall of 25 °C and ca. 3500 mm, respectively.

The composition of the plant community at this sampling site was well known, as monitoring of the forest dynamics has been carried out since 2006 [11]. The evaluation of the composition in this study was performed using the same methodology as previously described [11]. Briefly, a transect was created along the central axis of the site (one meter wide) to evaluate the abundance of all seedlings and plants of woody species with diameters above 1 cm at breast height. The size of the site was 20 m × 10 m (i.e., 200 m^2^). Among the thirteen woody plant species in this plant community (Figure 1B), six of them were representatives of the most common species that were sampled for this study: the island-endemic shrub to small tree *Ixora mooreensis* (Rubiaceae, hereafter named IXO), the native small tree *Cyclophyllum barbatum* (Rubiaceae, hereafter named CYC), the Polynesian-introduced large tree *Inocarpus fagifer* (Fabaceae, hereafter named INO) and small tree *Syzygium malaccense* (Myrtaceae, hereafter named SYZ), and the European-introduced small tree *Miconia calvescens* (Melastomataceae, hereafter named MIC) and large tree *Spathodea campanulata* (Bignoniaceae, hereafter named SPA).

In particular, soil samples were taken for each plant species from seedlings (four replicates were taken directly from roots) and trees (three replicates were taken close to trunks at a depth of 2–3 cm). Metal pliers were used to take samples, and they were cleaned with baths of bleach (40%), ethanol (70%), and sterile water between each sample to avoid contamination. Samples were either placed in DNA/RNA shield (250 mg) (ref. ZR1100-50) for metabarcoding analyses or in 50 mL falcon tubes (15 g) for metabolomics. For metabolomics, three root samples were also taken from different MIC trees in order to differentiate the endo- (metabolites produced within roots by MIC) and exometabolomes (metabolites only found outside roots and potentially produced by MIC and rhizospheric organisms). Metabolomic samples were then freeze-dried for 48 h and stored at −80 °C before subsequent processing.

### 2.2. DNA Extraction, PCR, and Sequencing

DNA extractions were performed using a ZymoBIOMICS DNA Miniprep Kit (ref. D4300) according to the manufacturer’s protocol. The variable V3V4 loops (341F: 5′-CCTACGGGNGGCWGCAG-3′; 805R: 5′-GACTACHVGGGTATCTAATCC-3′) [16] of the 16S rRNA gene of the bacterial communities were amplified and sequenced. In addition, the 18S rRNA gene of the eukaryotic communities (microeukaryotes and metazoans) was amplified and sequenced using the variable V4 loop (TAReuk454FWD1: 5′-CCAGCASCYGCGGTAATTCC-3′; TAReukREV3: 5′-ACTTTCGTTCTTGATYRA-3′) [17]. Paired-end sequencing (250 bp) was performed at McGill University (Génome Québec Innovation Centre, Montréal, Canada) using the v2 chemistry of the MiSeq system (Illumina). Raw sequence data are available in the Sequence Read Archive database (BioProject ID PRJNA945393).

### 2.3. Sequence Analyses

Trimmomatic [18] (default parameters, except MINLEN = 100) and the DADA2 package [19] (truncLen = c(245,245); maxN = 0; maxEE = c(2,2); truncQ = 2) were used to define the amplicon sequence variant (ASV) and computed taxonomic affiliations using the Silva database (release 138, December 2019). The taxonomic affiliations were used to generate three datasets: bacteria, microeukaryotes, and metazoa. Each dataset was filtered for singletons. Lastly, the tax_glom function was used to obtain the abundances at different taxonomic ranks (from genus to phylum).

### 2.4. Extraction of Metabolites and UHPLC-HRMS Profiling

For sample extraction, HPLC-grade acetonitrile (ACN) (Honeywell Riedel de Haen™), HPLC-grade isopropanol (propan-2-ol), formic acid RS for LC-MS (Carlo Erba, Val de Reuil, France), HPLC-grade water (VWR™), and HPLC-grade methanol (MeOH) (Fontenay-sous-Bois, France) were used. For the UHPLC-HRMS analysis, LC-MS-grade water (VWR™, Fontenay-sous-Bois, France), LC-MS-grade acetonitrile, and formic acid RS for LC-MS (Carlo Erba, Val de Reuil, France) were used. The extraction was adapted from the thesis work of Hikmat Ghosson [20]. First, 5 g of sample soil from each plant was placed in a new 50 mL falcon tube. Next, 15 mL of solvent S1 (ACN/isopropanol (70/30)) was added, vortexed, and shaken for 10 min in a bench mixer at 2500 RPM. The samples were then centrifuged (Allegra X-30R centrifuge, Beckman Coulter, Indianapolis, IN, USA) for 10 min at 4500 RPM at 10 °C. Then, 10 mL of the supernatant was stored in a new 50 mL falcon tube, 15 mL of solution S2 (H_2_O/MeOH (20/80) + 1% formic acid) was added to the sample, and the previous homogenization and centrifugation steps were repeated to remove 10 mL of supernatant. The 20 mL of supernatant was homogenized, and 10 mL was added to a hemolysis tube for dry evaporation (Genevac EZ-2 plus HCl compatible, SP Scientific, Warminster, PA, USA). The obtained extract was then absorbed in MeOH at a concentration of 0.5 mg/mL and injected into a UHPLC-DAD-MS Q Exactive Plus Orbitrap.

All the organic extracts were profiled using a Vanquish UHPLC system from ThermoScientific (Waltham, MA, USA) that was equipped with a Q Exactive™ Plus Orbitrap mass spectrometer with an electrospray ionization source and a diode array detector (DAD Vanquish, ThermoFisher scientific (Waltham, MA, USA). Metabolite separation was performed on a C18 UHPLC column (Luna^®^ Omega 1.6 µm Polar C18 100 A LC Column, 100 × 2.1 mm, Phenomenex, CA, USA). The chromatographic separation was carried out with a binary gradient. The column oven was set at 30 °C with an injection volume of 3 µL. The eluents used for the mobile phase were (A) water with 0.1% formic acid and (B) acetonitrile with 0.1% formic acid. The flow rate was 400 µL/min. The gradient conditions were 0–2.5 min, 2% (B); 2.5–17 min, 100% (B) with a linear gradient; 17–21 min, 100% (B) in isocratic mode; 21–23 min, 2% (B) with a linear gradient; and 23–25 min, 2% (B) in isocratic mode for column equilibration. The total run time was 25 min. The extracts were randomly injected, alternating with the quality control sample injections every six samples. The quality control samples were prepared by pooling equal volumes from all soil samples. Ultraviolet-visible (UV-Vis) detection was performed for wavelengths between 200 and 800 nm. Ionization was performed in the positive and negative electrospray modes, and mass detection was computed with a full scan MS window of 100–1500 *m*/*z* (resolutions of 70,000 and 17,500 in MS/MS mode and in MS mode, respectively) for a mass of 200 Da (Dalton). The capillary temperature was 320 °C, and the voltage applied to the nebulizer needle was 3.2 kV. A mass calibration was realized before starting the analysis. MS/MS fragmentations were performed on the five strongest ions, with three normalized collision energies of 20, 30, and 40 eV. Isotope fragmentation exclusion was activated in order to achieve a robust spectral similarity network.

### 2.5. Data Processing for Metabolomics

The raw data from the extracts obtained using UHPLC-HRMS in the positive and negative modes were converted into mzXML files with the Proteowizard v3.0 19202 software [21]. Data processing with W4M [22,23] was performed in 7 key steps: the identification of peaks for each sample, the clustering of similar peaks across all samples, retention time correction, the re-clustering of similar peaks across samples, peak integration followed by annotation, the removal of ions from blanks and the background, statistical analysis, and finally data visualization. A step verifying the quality of the generated matrix was performed at the end of the treatment. Among these steps, some fundamental parameters are listed as an example for the negative ionization mode: an interval of the *m*/*z* value for peak picking of 0.001, a signal-to-noise ratio threshold of 10, a group bandwidth of 5, and a minimum fraction of 0.011. The matrices generated from both ionization modes were exported as text files for subsequent analyses.

### 2.6. Multivariate and Statistical Analyses

All statistical analyses were performed using R v4.0.4 (R Development Core Team, 2008).

The {phyloseq} R package and the ggrare function were used to compute rarefaction curves of species richness for the metabarcoding dataset. The rarefy_even_depth function was used to subsample both the metabarcoding and metabolomic datasets. The alpha diversity metrics (Chao1 and Shannon) were obtained using the estimate_richness function. We performed one-way ANOVA or non-parametric Kruskal–Wallis tests (when the normality of residuals was rejected (Shapiro test)) to compare alpha diversity metrics between plant species. When the ANOVA or Kruskal–Wallis tests were significant, we computed pairwise comparisons between group levels (post hoc analyses) using pairwise *t*-tests or Dunn tests, respectively.

Principal coordinate analyses (hereafter named PCoA) (pcoa, {vegan}) were computed to describe the compositions of the ASVs and metabolites between samples using Bray–Curtis dissimilarities (vegdist, {vegan}). A permutational multivariate analysis of variance (hereafter named PERMANOVA) was used to compare the rhizosphere compositions between plant species and between seedlings and trees using 999 permutations (adonis, {vegan}). When a PERMANOVA was significant for plant species, we used an indicator value index (hereafter named IndVal) and 999 permutations (multipatt, {indicspecies}) [24] to identify specific taxa associated with the different plant species. The relative abundances and the heatmap2 function ({gplots}) were used to compute heatmaps of the specific ASVs.

A supervised partial least square-discriminant analysis (hereafter named PLS-DA) was performed between MIC and the other plant species at the tree stage in order to identify MIC-specific endo- and exometabolites (within and outside roots, respectively) using the R packages ggplot2, mixOmics, and factoextra. This analysis maximized the separation between the two groups and thus highlighted the importance of each variable in the projection thanks to the estimation of the variable importance in projection (hereafter named VIP) scores. In order to keep the most discriminant VIP, a second PLS-DA was computed with the VIPs with a score above 1. The new VIPs greater than 1 were kept, and a permutation test and a double cross-validation test (2CV) were performed to validate the PLS-DA model. Manual checking of the VIPs was processed using the MzMine2 software [25,26,27,28]. First, we performed an ion extraction at the defined retention time and checked the scan number and the gaussian that formed the peak associated with the metabolite of interest as well as its isotopic mass. Then, the VIPs were statistically compared using a non-parametric Wilcoxon test (wilcox.test, {stats}) between MIC and the other plant species. Lastly, correlation analyses between the metabarcoding and the exometabolome of MIC (100 best VIPs) were computed using DIABLO (DIABLO.test {mixOmics}) [29].

### 2.7. Phylogenetic Analyses

We performed BLASTn searches of MIC-associated taxa (Cryptomycota, Dorylaimida, and the ten most abundant bacterial ASVs found in MIC trees) on NCBI (non-redundant nucleotide collection). We kept the best hits to compute phylogenetic reconstructions. Reference sequences of Cryptomycota [30] and Dorylaimida [31] were added to the alignments of microeukaryotes and metazoans, respectively. Sequences were aligned using MAFFT [32] and trimmed at each extremity. GBlocks [33] was used to automatically remove the poorly aligned and highly variable regions of the alignments. Maximum-likelihood (ML) trees were computed with IQ-TREE v1.3.8. The best model was selected with the Bayesian information criterion [34]. An ultrafast bootstrap procedure with 1000 replicates was used to validate the trees [35].

### 2.8. Metabolite Annotation

Molecular networks were generated in negative modes via MzXml files using the online workflow on GNPS (http://gnps.ucsd.edu) (accessed on 19 February 2023) [36]. MS/MS spectra were filtered by choosing only the top six peaks in a +/−50 Da window throughout the spectrum. Data were clustered for some analyses using MSCluster (parent mass tolerance and MS/MS fragment ion tolerance of 0.02 Da) to create consensus spectra [37]. The edges between two nodes in a network were kept if each of them appeared in each other’s respective top 10 most similar nodes. The library spectra were filtered similarly to the input data. All matches kept between the library spectra and a network were required to show a cosine score above 0.7 and at least six matched peaks. The generated similarity networks were compared to a set of databases proposed by GNPS (CASMI Spectral Library, GNPS library).

All the spectral similarity networks generated with GNPS were imported into Cytoscape v3.8.2 [38]. In a first step, the spectral similarity networks, including the VIPs, were isolated for each ionization mode. For each mode (positive and negative ionization), all the metadata were merged and harmonized between the networks to obtain a unique network of similarity. For this, each MS and MS/MS of each ion from the GNPS networks were compared two by two in order to be validated as being the same. Following this, pie charts representing the proportion of each variable were harmonized by consensus into an average value from the two previous studies. The connections to the other nodes were in turn harmonized into a mean cosine score derived from the values obtained with GNPS. Before validating this step, the ions were checked to ensure they did not belong to the background and were not artifacts related to an error during data processing. The AllegroLayout representation mode was used to organize the spatial arrangement of the arrays in Cytoscape.

Molecular formulae of significant features were calculated using Sirius 5.6.2 [39]. In order to reduce the number of potential candidates, various parameters were used, such as the element selection exclusively including C, H, and O. The isotopic ratio tolerance was set to 20%, and the mass tolerance was fixed at 5 ppm. Only natural product databases of plants were selected. A list of compounds sorted according to the score value of the match was obtained. This value encompassed the uncertainty of the isotopic pattern score, the accurate mass, and the experimental MS/MS fragmentation mirrored in in silico matches. Chemical classes were kept for identified features with scores above 5 and structures with scores above 70% for similarity.

## 3. Results

### 3.1. Plant Community at the Sampling Site

The sampling site was located in the Opunohu valley on the island of Mo’orea (Figure 1A). The plant community was composed of 13 woody plant species (Figure 1B). Six of them were selected to study the rhizosphere using metabarcoding and metabolomics: *Cyclophyllum barbatum* (CYC), *Inocarpus fagifer* (INO), *Ixora mooreensis* (IXO), *Miconia calvescens* (MIC), *Spathodea campanulata* (SPA), and *Syzygium malaccense* (SYZ).

### 3.2. Dominant Taxa Associated with the Six Plant Species

**Figure 2:**
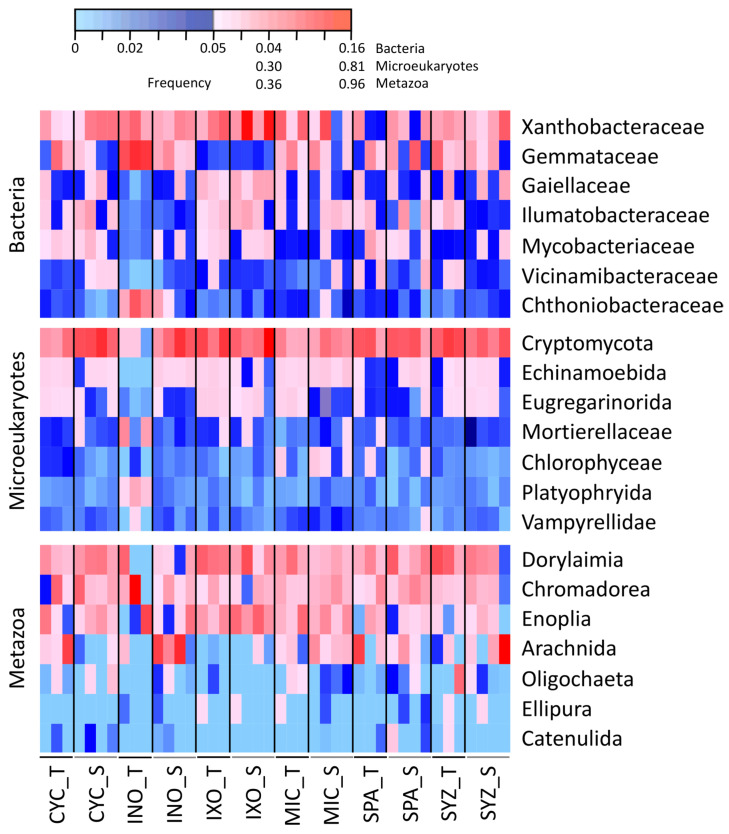
Dominant families at the sampling site for bacteria, microeukaryotes, and metazoans. CYC: *Cyclophyllum barbatum*, INO: *Inocarpus fagifer*, IXO: *Ixora mooreensis*, MIC: *Miconia calvescens*, SPA: *Spathodea campanulata*, SYZ: *Syzygium malaccense*. T: tree. S: seedling.

The whole soil communities associated with the six plant species were sequenced using the 16S and 18S rRNA genes from 24 seedlings and 18 trees. On average, each sample contained 24,209 sequences representing 311 ASVs (Figure S1 and Tables S1–S4). Xanthobacteraceae, Cryptomycota, and Dorylaimia represented the most abundant and common families of bacteria, microeukaryotes, and metazoans, respectively (Figure 2). Moreover, metabolomics was used to describe the metabolite diversity within the same samples according to the positive (Table S5) and negative (Table S6) ionization modes. Three root samples were also taken from MIC trees to differentiate endo- (metabolites produced within roots by MIC) and exometabolomes (metabolites only found outside roots and potentially produced by MIC and rhizospheric organisms).

### 3.3. Seedling Effects on Soil Diversity

**Figure 3:**
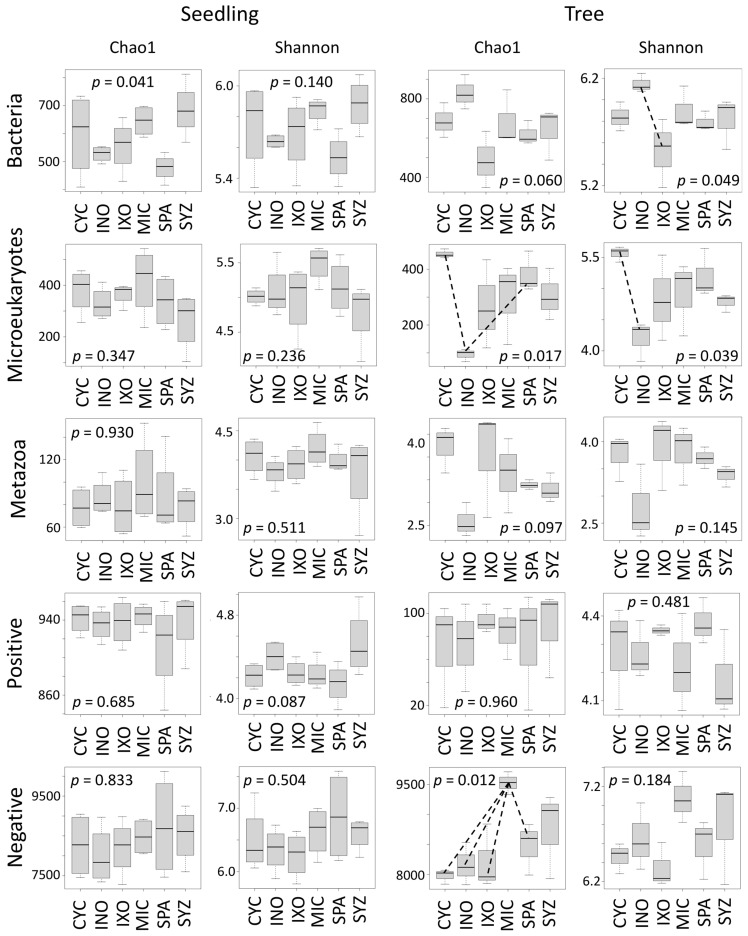
Shannon and Chao1 metrics in seedling and tree stages. CYC: *Cyclophyllum barbatum*, INO: *Inocarpus fagifer*, IXO: *Ixora mooreensis*, MIC: *Miconia calvescens*, SPA: *Spathodea campanulata*, SYZ: *Syzygium malaccense*. Dashed lines indicate significant pairwise comparisons.

**Figure 4:**
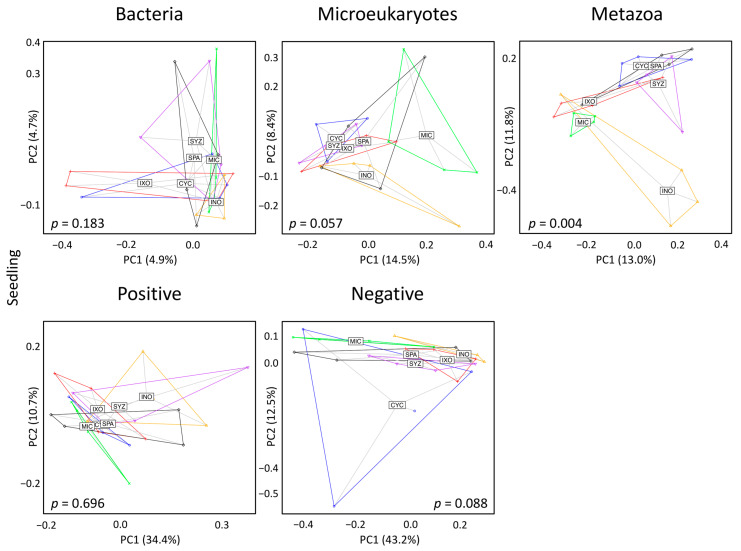
Principal coordinate analysis in the seedling stage. Bray–Curtis dissimilarities between samples were used for these analyses. Each dot corresponds to one sample. Labels of plant species are displayed at the barycenters of dots. CYC: *Cyclophyllum barbatum*, INO: *Inocarpus fagifer*, IXO: *Ixora mooreensis*, MIC: *Miconia calvescens*, SPA: *Spathodea campanulata*, SYZ: *Syzygium malaccense*.

**Figure 5:**
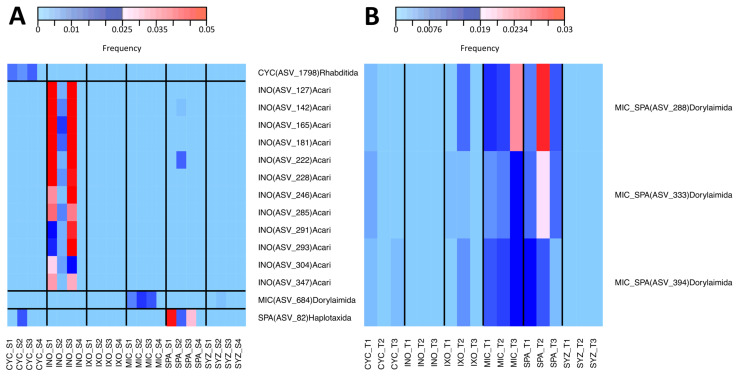
Heatmap of specific metazoan ASVs. Only ASVs specific to a single plant species or to both MIC and SPA are shown for the seedling and tree stages, respectively. For each ASV, the name of the associated plant species is indicated as well as its taxonomic annotation. CYC: *Cyclophyllum barbatum*, INO: *Inocarpus fagifer*, IXO: *Ixora mooreensis*, MIC: *Miconia calvescens*, SPA: *Spathodea campanulata*, SYZ: *Syzygium malaccense*. ASV: amplicon sequence variant. (**A**) Seedling stage. S: seedling. (**B**) Tree stage. T: tree.

First, we analyzed whether the six plant species had different effects on the soil diversity in the seedling stage using the Shannon and the Chao1 metrics (Table S7), but a significant effect was found only for the Chao1 index of bacteria (Figure 3). Moreover, no effect was detected on soil composition (beta diversity) using PCoA and PERMANOVA, except for metazoans (Figure 4). In order to explain the significant effect found for metazoans, we used IndVal to identify the specific metazoan taxa associated with the six plant species. Significant associations were identified for 27 ASVs (Table S8). In particular, CYC was associated with Rhabditida (nematodes), INO was associated with Acari, and SPA was associated with Haplotaxida (Oligochaeta) (Figure 5A). Only one ASV (ASV_684, Dorylaimida (nematodes)) was specific to MIC.

### 3.4. Developmental Effect

Then, we compared the rhizosphere compositions of seedlings and trees to identify potential developmental effects. Using a PERMANOVA, significant developmental effects were found for INO on bacterial composition; for CYC, INO, MIC, and SYZ on microeukaryotes; and for INO, MIC, and SYZ on metazoans (Table 1). As a consequence, INO had a strong developmental effect on the three types of assemblages (bacteria, microeukaryotes, and metazoans). In addition, while MIC and SYZ had different effects on microeukaryotes and metazoans during development, CYC only had different effects on microeukaryotes. No developmental changes were highlighted for the other plant species and the metabolomic dataset.

### 3.5. Tree Effects on Soil Diversity

Significant effects were found in the tree stage using the Shannon index for bacteria and microeukaryotes but not for metazoans (Figure 3). In addition, significant effects were identified for microeukaryotes and the negative mode using the Chao1 index. In particular, this analysis highlighted that MIC contained higher metabolite richness than the other plant species.

**Figure 6:**
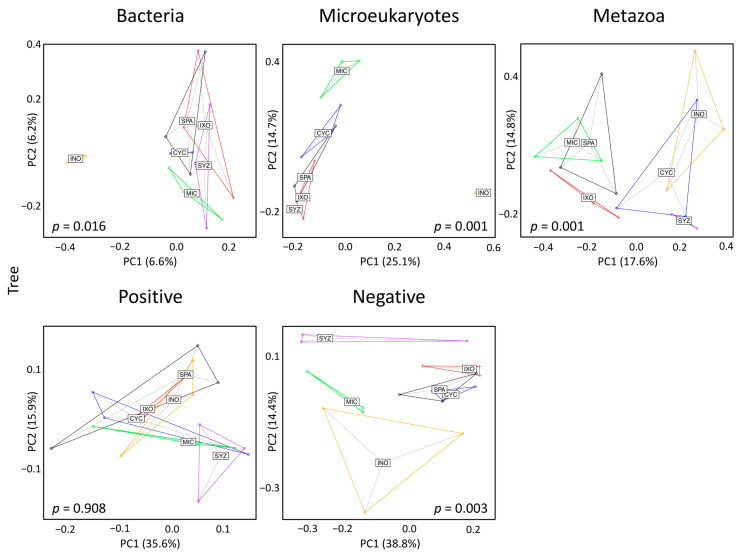
Principal coordinate analysis in the tree stage. Bray–Curtis dissimilarities between samples were used for these analyses. Each dot corresponds to one sample. Labels of plant species are displayed at the barycenters of dots. CYC: *Cyclophyllum barbatum*, INO: *Inocarpus fagifer*, IXO: *Ixora mooreensis*, MIC: *Miconia calvescens*, SPA: *Spathodea campanulata*, SYZ: *Syzygium malaccense*.

Significant effects were also obtained for the three types of taxa and the negative mode when the beta diversity was analyzed using PCoA and PERMANOVA (Figure 6). The significances seemed to be mostly due to INO, but the effects were still significant when INO was discarded from the datasets of microeukaryotes, metazoans, and the negative mode but not the dataset of bacteria (Figure S2). In particular, MIC formed an isolated cluster for microeukaryotes and the negative mode.

### 3.6. Specific Taxa of Each Plant Species in the Tree Stage

**Figure 7:**
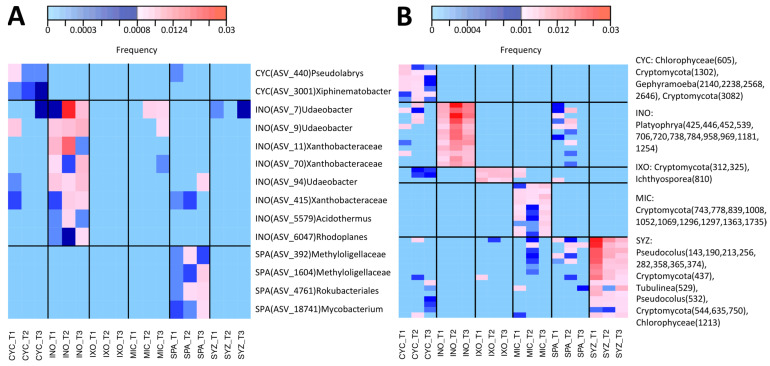
Heatmap of specific bacterial and microeukaryote ASVs in the tree stage. Only ASVs specific to a single plant species are shown. For each ASV number, the name of the associated plant species is indicated as well as its taxonomic annotation. CYC: *Cyclophyllum barbatum*, INO: *Inocarpus fagifer*, IXO: *Ixora mooreensis*, MIC: *Miconia calvescens*, SPA: *Spathodea campanulata*, SYZ: *Syzygium malaccense*. T: tree. ASV: amplicon sequence variant. (**A**) Bacterial ASV. (**B**) Microeukaryote ASV.

IndVal was used to identify specific taxa associated with the six plant species in the tree stage (Table S8). The significant effect on bacteria was mostly due to INO. As a consequence, most of the 15 significant ASVs were associated with INO and were rhizobia. None were specific to MIC. Among them, *Candidatus Udaeobacter* (Chthoniobacterales), Xanthobacteraceae (rhizobiales), and *Acidothermus* (Frankiales) were significantly associated with INO (Figure 7A). Significant associations were found for 19 metazoan ASVs (Table S8). Most belonged to Dorylaimida, but none were specific to a single plant species. However, three of them were specific to both MIC and SPA (Figure 5B). Moreover, 109 significant associations were identified between microeukaryotes and plant species. Among them, INO was associated with *Platyophrya* (ciliates), CYC was associated with *Gephyramoeba*, SYZ was associated with *Pseudocolus* (fungi), and IXO was associated with Cryptomycota (Figure 7B). Ten ASVs were specific to MIC and belonged to the Cryptomycota family.

### 3.7. Phylogenetic Analyses of MIC-Associated Taxa

**Figure 8:**
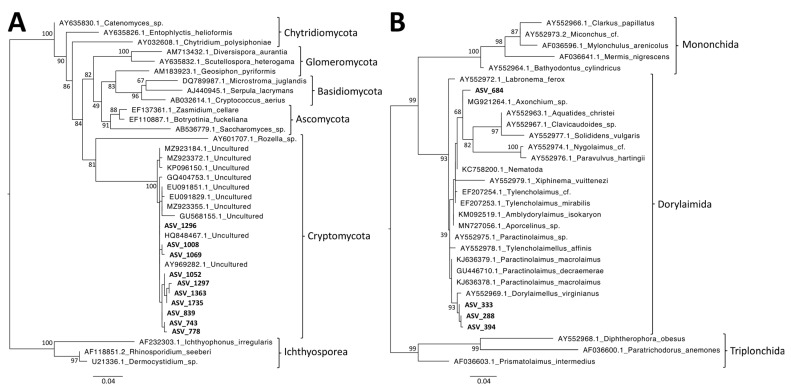
Maximum-likelihood phylogenetic trees. Numbers are ultrafast bootstraps (%) of the main nodes. ASV: amplicon sequence variant. (**A**) Significant Cryptomycota ASVs. The tree was rooted using Ichthyosporea. The ASVs used in this figure were all significantly associated with *Miconia calvescens*. (**B**) Significant Dorylaimida ASVs. The tree was rooted using Triplonchida. ASV_684 was significantly associated with MIC. ASV_288, ASV_333, and ASV_394 were significantly associated with both *Miconia calvescens* and *Spathodea campanulata*.

As significant associations were identified between MIC and soil taxa (microeukaryotes and metazoans) in the seedling and tree stages, phylogenetic analyses were computed with the best hits from the NCBI database to better describe their diversity. While microeukaryotes were similar to uncultured Cryptomycota (Figure 8A), metazoans were close to *Axonchium* sp. (MG921264.1) and *Dorylaimellus virginianus* (AY552969.1) (Figure 8B). Despite a lack of significant associations with bacteria, a similar phylogenetic analysis was computed to describe the diversity of the ten most abundant ASVs of MIC trees (Figure S3). The 10 ASVs were similar to cultured strains of *Catellatospora tritici* (NR_181780.1), *Desulfovibrio subterraneus* (NR_179335.1), *Embleya scabrispora* (NR_112597.1), *Gemmata palustris* (NR_181623.1), *Limisphaera ngatamarikiensis* (NR_134756.1), *Micromonospora narathiwatensis* (NR_041256.1), *Patulibacter brassicae* (NR_153670.1), *Rhodoplanes azumiensis* (NR_159239.1), and *Solirubrobacter ginsenosidimutans* (NR_108192.1).

### 3.8. Specific Metabolites of MIC and Links with Rhizospheric Organisms

Because the identification of specific metabolites requires a rigorous verification of each peak, this analysis was only performed for MIC. A two-step PLS-DA was used to identify specific metabolites of MIC (see Materials and Methods). The obtained model was robust, according to the CER (0.011), *p*-value (0.002), and miss-classification rate (0%). At this step, 1300 VIPs (MIC-specific metabolites) were obtained (value > 1), and among them 40.9% were endometabolites (present in MIC roots) and 59.1% were exometabolites (only present in the rhizosphere).

**Figure 9:**
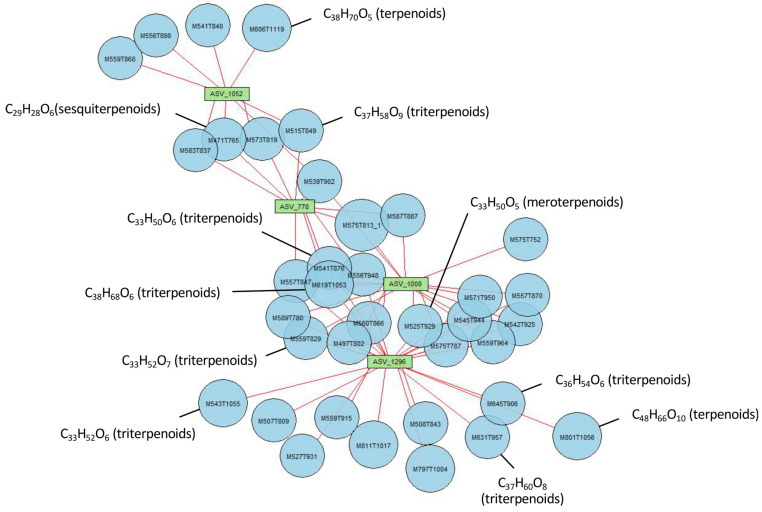
Correlation analyses between exometabolites and microeukaryotes. Only correlations above 98% were used to compute the network. Red lines between nodes indicate positive correlations.

The 25 best endometabolite VIPs were manually checked, and 15 of them were annotated using Sirius and MzMine2 (Table S9). Most belonged to the terpenoid class (Figure S4), except five, which were benzenoids, a flavonone, and a phenolic acid. The 100 best exometabolite VIPs were also manually checked and were then compared to the metabarcoding dataset in order to identify correlations between metabolite and taxa abundances. While no correlations were obtained between the exometabolites and abundances of both bacteria and metazoans, several links were highlighted with microeukaryotes. In particular, the highest correlations (above 98%) were found between four ASVs (ASV_778, ASV_1008, ASV_1052, and ASV_1296) and 37 exometabolites (Figure 9). All ASVs were affiliated with Cryptomycota, and 9 out of 37 metabolites were classified as terpenoids (Table S9).

## 4. Discussion

### 4.1. Trees Had Stronger Effects Than Seedlings on Soil Diversity

Our study revealed a weak effect of seedlings on soil diversity in comparison to trees. This result was expected and might be explained by a short influence in terms of time period as well as by the small rhizosphere surfaces of seedlings. Similar results were already obtained, for example, for arbuscular mycorrhizal fungi associated with the large tree *Swietenia macrophylla* (Meliaceae) [40]. Although our analyses highlighted the stronger effect of trees on soil diversity, we did not identify significant differences between seedlings and trees for each plant species and each dataset when we compared the matrix compositions using a PERMANOVA (Table 1). This observation may reflect a lack of statistical power due to a limited number of replicates. This number was set in this study to favor the use of a diverse set of data (metabarcoding and metabolomics) to exhaustively describe the soil diversity in order to identify the most important compartments for future analyses.

### 4.2. Inocarpus fagifer Strongly Influenced Assemblages of Bacteria, Microeukaryotes, and Metabolites

Among the six plant species, INO had the most important effect in the seedling stage and especially in the tree stage. INO is a legume that belongs to the Fabaceae family and the Faboideae sub-family [41]. This group of plants may live in symbiosis with bacteria named rhizobia. Legume plants produce flavonoids that attract rhizobia and favor the release of bacterial symbiotic factors [42]. As a consequence, it was not surprising to observe a strong impact on bacterial assemblages. The significant effects observed on microeukaryotes might be the result of cascading effects within the food network. Indeed, bacterial assemblages might favor the presence of bacteriophages such as the ciliate *Platyophrya* [43] (Figure 7B).

### 4.3. Miconia calvescens Strongly Influenced Assemblages of Microeukaryotes and Metabolites

Although no significant associations were identified between MIC and bacteria, a phylogenetic analysis revealed that the 10 most abundant ASVs of MIC trees were similar to cultured strains, and several were isolated from tropical and/or warm sites (among them, some strains were described as thermophilic) [44,45,46] (Figure S3). This suggested that the most abundant ASVs of MIC trees might be adapted to the tropical climate of Mo’orea.

In contrast, the specific effects of MIC on the composition of microeukaryotes and metabolites (negative mode) were visible in the PCoA (Figure 6) and were even larger when the INO samples were removed from the dataset (Figure S2). In particular, MIC showed specific interactions with several Cryptomycota (Figure 7B), and all of them were close to uncultured strains that were already identified in terrestrial soil [47,48,49,50] or freshwater sediment [51]. However, Cryptomycota were discovered recently; thus, their ecology is mostly unknown, even if it was proposed that they are likely parasites [52]. Moreover, even though we did not identify a specific effect on metazoans, MIC and SPA (two European-introduced and invasive trees in French Polynesia) harbored similar metazoan assemblages. Both plant species were significantly associated with Dorylaimida, which are nematodes that are mostly found in moist soils around plant roots [53]. Several genera are predaceous, and many are free-living, feeding on bacteria and microeukaryotes. The blastn and phylogenetic analyses highlighted that MIC ASVs are close to *Axonchium* sp. and *Dorylaimellus virginianus*, which were classified as plant feeders and possibly omnivorous and hyphal feeders, respectively [54]. The biological control of MIC was already tested using fungi [55], coleoptera [56], and also plant-feeding nematodes [57]. Thus, it might be important to test the nature of the interactions between Dorylaimida and the six plant species. At this step, it was not possible to determine the ecological roles of Cryptomycota and Dorylaimida, but future studies should test if they influence MIC fitness.

Another striking observation was the high metabolite richness associated with MIC trees (Figure 3, Chao1 index). This diversity might be composed of chemical weapons that favor MIC fitness [13], but additional studies must be performed in the future to elucidate the nature and the role of these compounds. In particular, many terpenoids composed the endometabolome of MIC, and correlation analyses revealed that several terpenoids from the exometabolome were correlated with microeukaryotes close to Cryptomycota. Both observations suggest that terpenoids were produced by MIC and that they favored the presence of Cryptomycota. Indeed, terpenoids might be involved in communication between plants and fungi [58]. However, we cannot reject the hypotheses that exoterpenoids were directly produced by Cryptomycota or other rhizospheric organisms or that endoterpenoids were synthesized by endophytic fungi [59]. The presence of meroterpenoids reinforces these hypotheses [60]. In addition, terpenoids are not only molecules involved in communication; they are also involved in inhibitory activities against bacteria and fungi [59]. As a consequence, more studies are needed in the future to identify the producers of these metabolites, the role of terpenoids, and whether they are linked to MIC fitness. In particular, future studies should be conducted on MIC trees using more replicates from more sites and focusing on the most important datasets represented by microeukaryotes, metazoans, and metabolites identified with the negative ionization mode. Isolations of Cryptomycota and Dorylaimida should be also performed to estimate their roles in MIC fitness and to determine whether they might be used as management tools.

### 4.4. Improvements Made by This Study

Most studies of the rhizosphere have concerned bacterial and fungal diversity. However, the rhizosphere is also composed of diverse communities of microeukaryotes and metazoans. To our knowledge, this was the first study that deeply revealed the soil diversity using metabarcoding in French Polynesia, and the significant links obtained with microeukaryotes and metazoans highlighted the importance of analyzing these compartments. In addition, most studies of metabolomics have focused on beta diversity analyses, but few of them have considered alpha diversity metrics [61]. The significant link obtained for MIC using the Chao1 index also emphasized the importance of using these metrics. Lastly, the combination of metabarcoding and metabolomics has rarely been used in previous studies of terrestrial soil [62,63,64,65]. Here, we showed that both methods offer exhaustive descriptions of soil diversity and that correlation analyses might shed light on putative metabolite producers.

## 5. Conclusions

**Figure 10:**
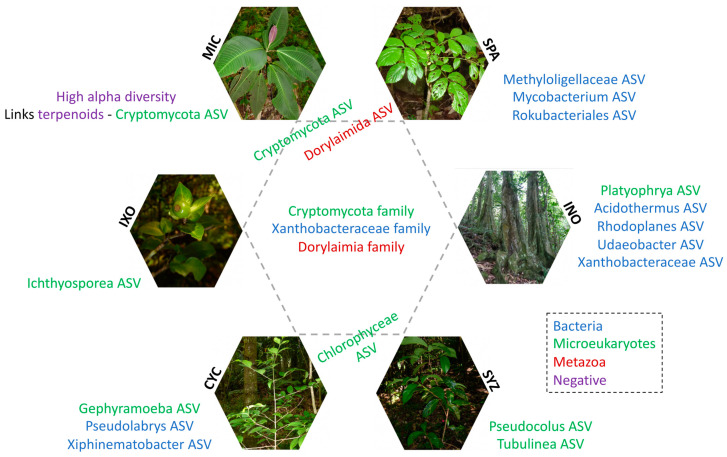
Specific and common features of the six plant species in the tree stage. CYC: *Cyclophyllum barbatum*, INO: *Inocarpus fagifer*, IXO: *Ixora mooreensis*, MIC: *Miconia calvescens*, SPA: *Spathodea campanulata*, SYZ: *Syzygium malaccense*. ASV: amplicon sequence variant. Specific features are indicated outside of the central hexagon. Common features are in the center of the central hexagon. Specific features shared between two (Chlorophyceae and Dorylaimida ASVs) or three (Cryptomycota ASVs) plant species are indicated at the limit of the central hexagon near the corresponding plant species. Features from bacteria, microeukaryotes, metazoans, and the negative mode are indicated in blue, green, red, and purple, respectively.

In conclusion, we compared the rhizosphere of *Miconia calvescens* with other plant species in order to describe how this invasive alien tree influences soil diversity and to identify chemicals and biomarkers. Our study highlighted that the trees had an effect on soil diversity and that the seedlings had more restricted effects. Despite common features between the six plant species in the tree stage (the families of Cryptomycota, Dorylaimia, and Xanthobacteraceae were the most shared), specific features were identified for each individual plant species (Figure 10). In particular, several terpenoids and Cryptomycota ASVs were specific to MIC and were defined as putative chemicals and biomarkers. The roles of these taxa remain unknown, but their presence was possibly linked to these metabolites, and we hypothesized that both features might be linked to MIC fitness.

## Figures and Tables

**Table 1:** Developmental effects on soil diversity. *p* values are indicated based on a PERMANOVA. Significant *p* values are in bold.

Species	Bacteria	Microeukaryotes	Metazoa	Positive	Negative
*Cyclophyllum barbatum*	0.973	**0.030**	0.342	0.691	0.190
*Inocarpus fagifer*	**0.030**	**0.029**	**0.050**	0.528	0.087
*Ixora mooreensis*	0.461	0.358	0.554	0.557	0.831
*Miconia calvescens*	0.808	**0.029**	**0.049**	0.706	0.219
*Spathodea campanulata*	0.278	0.504	0.100	0.638	0.402
*Syzygium malaccense*	0.757	**0.034**	**0.046**	0.490	0.153

## Data Availability

The datasets generated during the current study are available in the Sequence Read Archive repository under BioProject ID PRJNA945393.

## Supplementary Materials

The following supporting information can be downloaded at https://www.mdpi.com/article/10.3390/microorganisms11040832/s1, Figure S1: Rarefaction curves of metabarcoding sequences; Figure S2: Principal coordinate analysis without INO in the tree stage. Bray–Curtis dissimilarities between samples were used for these analyses. Each dot corresponds to one sample. Labels of plant species are displayed at the barycenters of dots. CYC: *Cyclophyllum barbatum*, IXO: *Ixora mooreensis*, MIC: *Miconia calvescens*, SPA: *Spathodea campanulata*, SYZ: *Syzygium malaccense*; Figure S3: Maximum-likelihood phylogenetic tree of dominant bacterial ASVs of MIC trees. The tree was rooted using an archaeon. Numbers are ultrafast bootstraps (%) reflecting clade support. The ASVs used in this figure were the 10 dominant ASVs of MIC in the tree stage. MIC: *Miconia calvescens*. ASV: amplicon sequence variant; Figure S4: Examples of spectral similarity networks of terpene biomarkers associated with their respective boxplots and annotations. MIC: *Miconia calvescens*; Table S1: Metadata and metabarcoding denoising; Table S2: Bacterial abundances and annotations. CYC: *Cyclophyllum barbatum*, INO: *Inocarpus fagifer*, IXO: *Ixora mooreensis*, MIC: *Miconia calvescens*, SPA: *Spathodea campanulata*, SYZ: *Syzygium malaccense*. S: seedling. T: tree. ASV: amplicon sequence variant; Table S3: Microeukaryote abundances and annotations. CYC: *Cyclophyllum barbatum*, INO: *Inocarpus fagifer*, IXO: *Ixora mooreensis*, MIC: *Miconia calvescens*, SPA: *Spathodea campanulata*, SYZ: *Syzygium malaccense*. S: seedling. T: tree. ASV: amplicon sequence variant; Table S4: Metazoan abundances and annotations. CYC: *Cyclophyllum barbatum*, INO: *Inocarpus fagifer*, IXO: *Ixora mooreensis*, MIC: *Miconia calvescens*, SPA: *Spathodea campanulata*, SYZ: *Syzygium malaccense*. S: seedling. T: tree. ASV: amplicon sequence variant; Table S5: Metabolites identified using the positive ionization mode. CYC: *Cyclophyllum barbatum*, INO: *Inocarpus fagifer*, IXO: *Ixora mooreensis*, MIC: *Miconia calvescens*, SPA: *Spathodea campanulata*, SYZ: *Syzygium malaccense*. S: seedling. T: tree. R: root; Table S6: Metabolites identified using the negative ionization mode. CYC: *Cyclophyllum barbatum*, INO: *Inocarpus fagifer*, IXO: *Ixora mooreensis*, MIC: *Miconia calvescens*, SPA: *Spathodea campanulata*, SYZ: *Syzygium malaccense*. S: seedling. T: tree. R: root; Table S7: Alpha diversity metrics. CYC: *Cyclophyllum barbatum*, INO: *Inocarpus fagifer*, IXO: *Ixora mooreensis*, MIC: *Miconia calvescens*, SPA: *Spathodea campanulata*, SYZ: *Syzygium malaccense*. S: seedling. T: tree; Table S8: Significant associations between plant species and soil taxa. CYC: *Cyclophyllum barbatum*, INO: *Inocarpus fagifer*, IXO: *Ixora mooreensis*, MIC: *Miconia calvescens*, SPA: *Spathodea campanulata*, SYZ: *Syzygium malaccense*. 1: presence of the taxa for the corresponding plant species. 0: absence. ASV: amplicon sequence variant; Table S9: Metabolite annotations.
