# Supplementary material for: Metabarcoding and Metabolomics Reveal the Effect of the Invasive Alien Tree Miconia calvescens DC. on Soil Diversity on the Tropical Island of Mo’orea (French Polynesia)
Source: Microorganisms. 2023 Mar 24;11(4):832. doi: 10.3390/microorganisms11040832 (PMC10144827; doi:10.3390/microorganisms11040832)
Supplement: Supplementary file 1 [file microorganisms-11-00832-s001.zip › Titles_of_supplementary_files_v2_marked.pdf]

Titles of supplementary files

Figure S1. Rarefaction curves of metabarcoding sequences.

Figure S2. Principal coordinate analysis without INO at the tree stage. Bray-Curtis dissimilarities between samples were used for these analyses. Each dot corresponds to one sample. Labels of plant species are displayed at the barycenter of dots. CYC: *Cyclophyllum barbatum*, IXO: *Ixora mooreensis*, MIC: *Miconia calvescens*, SPA: *Spathodea campanulata*, SYZ: *Syzygium malaccense*.

Figure S3. Maximum-likelihood phylogenetic tree of dominant bacterial ASV of MIC trees. The tree was rooted using an archaeon. Numbers are ultrafast bootstraps (%) reflecting clade support. The ASV used in this figure were the 10 dominant ASV of MIC at the tree stage. MIC: *Miconia calvescens*. ASV: amplicon sequence variant.

Figure S4. Examples of spectral similarity networks of terpenes biomarkers associated with their respective boxplot and annotation. MIC: *Miconia calvescens*.

Table S1. Metadata and metabarcoding denoising.

Table S2. Bacterial abundances and annotations. CYC: *Cyclophyllum barbatum*, INO: *Inocarpus fagifer*, IXO: *Ixora mooreensis*, MIC: *Miconia calvescens*, SPA: *Spathodea campanulata*, SYZ: *Syzygium malaccense*. S: seedling. T: tree. ASV: amplicon sequence variant.

Table S3. Microeukaryotes abundances and annotations. CYC: *Cyclophyllum barbatum*, INO: *Inocarpus fagifer*, IXO: *Ixora mooreensis*, MIC: *Miconia calvescens*, SPA: *Spathodea campanulata*, SYZ: *Syzygium malaccense*. S: seedling. T: tree. ASV: amplicon sequence variant.

Table S4. Metazoan abundances and annotations. CYC: *Cyclophyllum barbatum*, INO: *Inocarpus fagifer*, IXO: *Ixora mooreensis*, MIC: *Miconia calvescens*, SPA: *Spathodea campanulata*, SYZ: *Syzygium malaccense*. S: seedling. T: tree. ASV: amplicon sequence variant.

Table S5. Metabolites identified with the positive ionization mode. CYC: *Cyclophyllum barbatum*, INO: *Inocarpus fagifer*, IXO: *Ixora mooreensis*, MIC: *Miconia calvescens*, SPA: *Spathodea campanulata*, SYZ: *Syzygium malaccense*. S: seedling. T: tree. R: root.

Table S6. Metabolites identified with the negative ionization mode. CYC: *Cyclophyllum barbatum*, INO: *Inocarpus fagifer*, IXO: *Ixora mooreensis*, MIC: *Miconia calvescens*, SPA: *Spathodea campanulata*, SYZ: *Syzygium malaccense*. S: seedling. T: tree. R: root.

Table S7. Alpha diversity metrics. CYC: *Cyclophyllum barbatum*, INO: *Inocarpus fagifer*, IXO: *Ixora mooreensis*, MIC: *Miconia calvescens*, SPA: *Spathodea campanulata*, SYZ: *Syzygium malaccense*. S: seedling. T: tree.

Table S8. Significant associations between plant species and soil taxa. CYC: *Cyclophyllum barbatum*, INO: *Inocarpus fagifer*, IXO: *Ixora mooreensis*, MIC: *Miconia calvescens*, SPA: *Spathodea campanulata*, SYZ: *Syzygium malaccense*. 1: presence of the taxa for the corresponding plant species. 0: absence. ASV: amplicon sequence variant.

Table S9. Metabolite annotations.
