# Supplementary figures and images for: Metabarcoding and Metabolomics Reveal the Effect of the Invasive Alien Tree Miconia calvescens DC. on Soil Diversity on the Tropical Island of Mo’orea (French Polynesia)
Source: Microorganisms. 2023 Mar 24;11(4):832. doi: 10.3390/microorganisms11040832 (PMC10144827; doi:10.3390/microorganisms11040832)

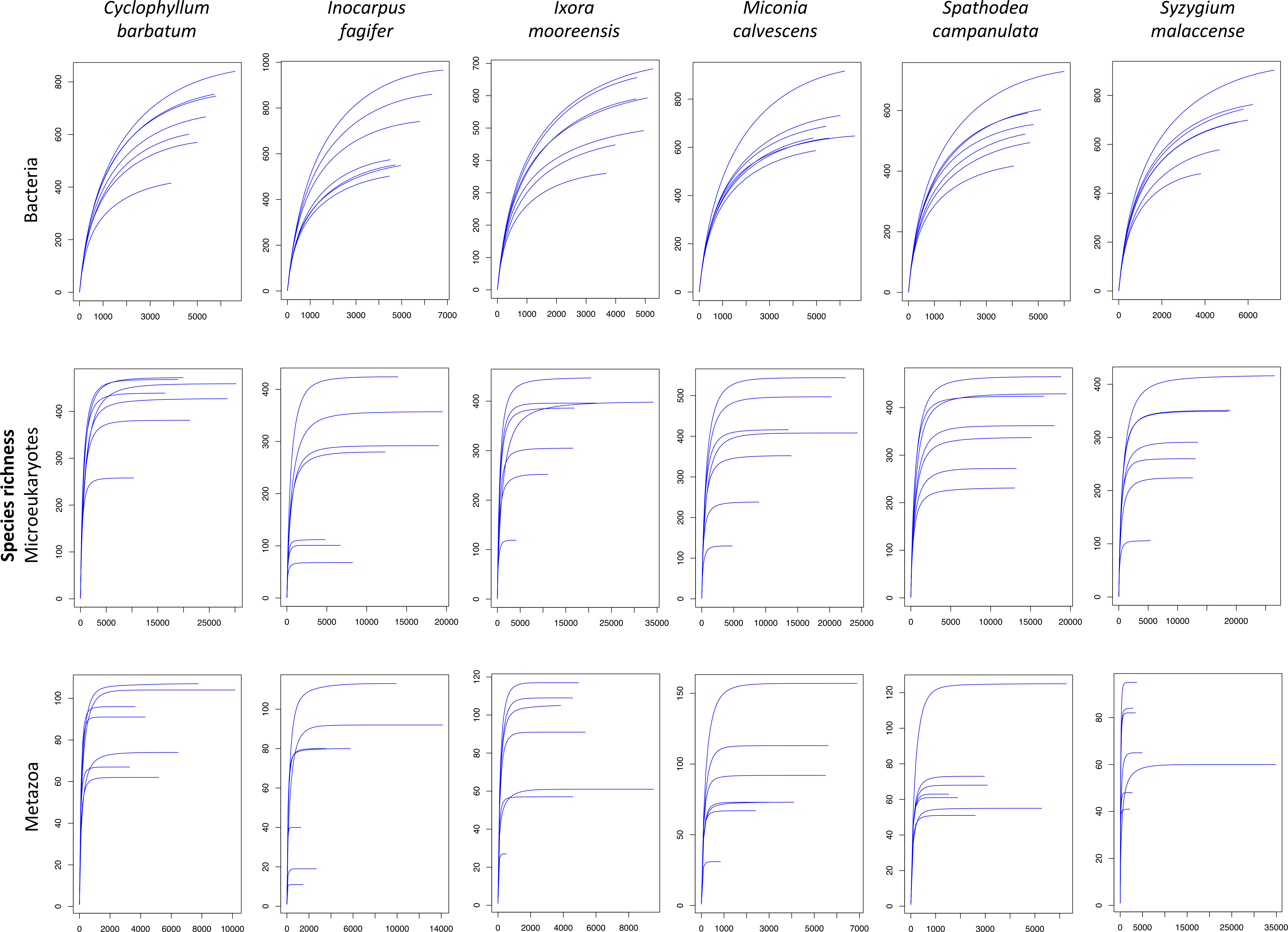

Supplement: Supplementary file 1 [file microorganisms-11-00832-s001.zip › Figure S1.tiff]

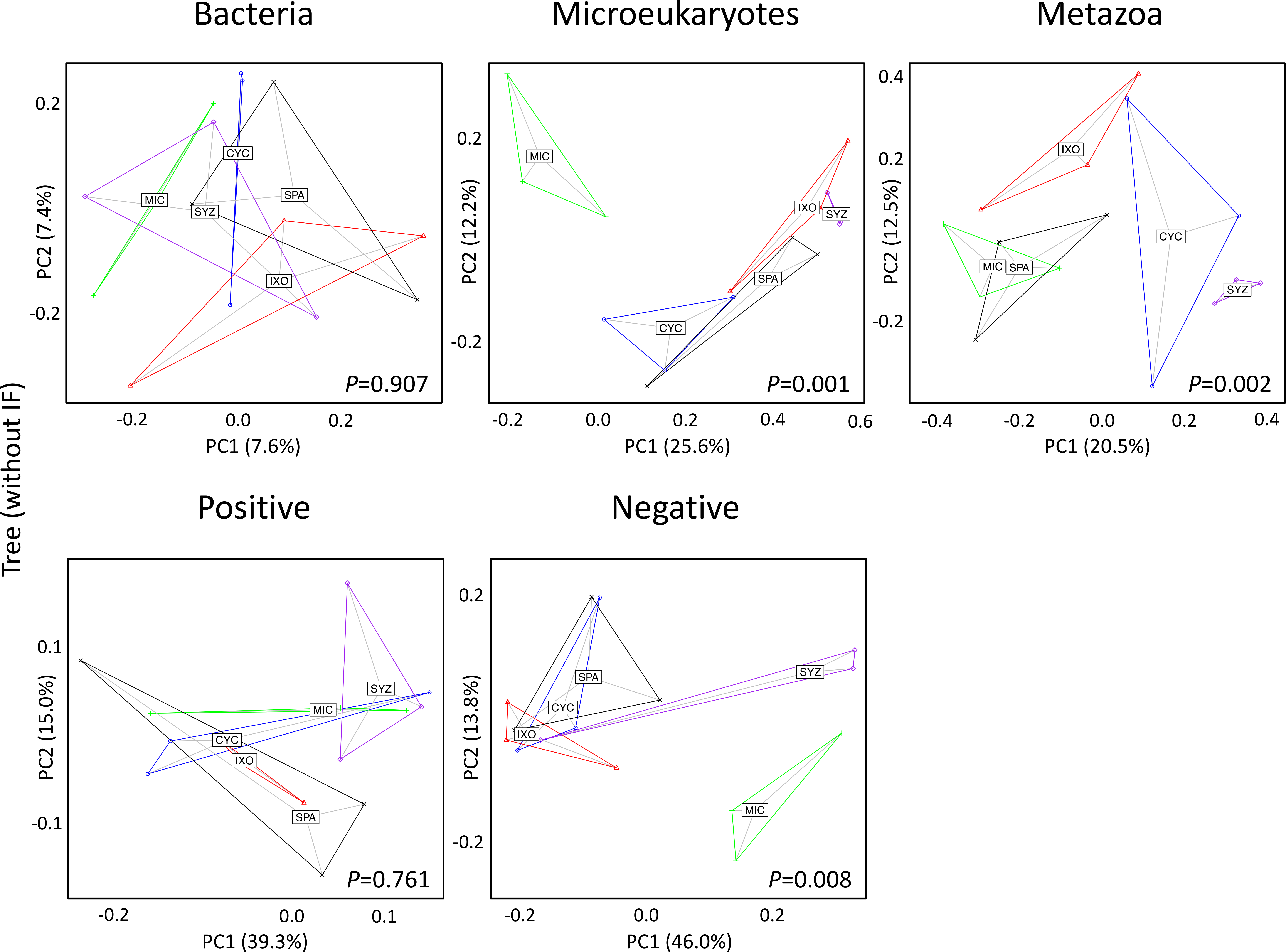

Supplement: Supplementary file 1 [file microorganisms-11-00832-s001.zip › Figure S2.tiff]

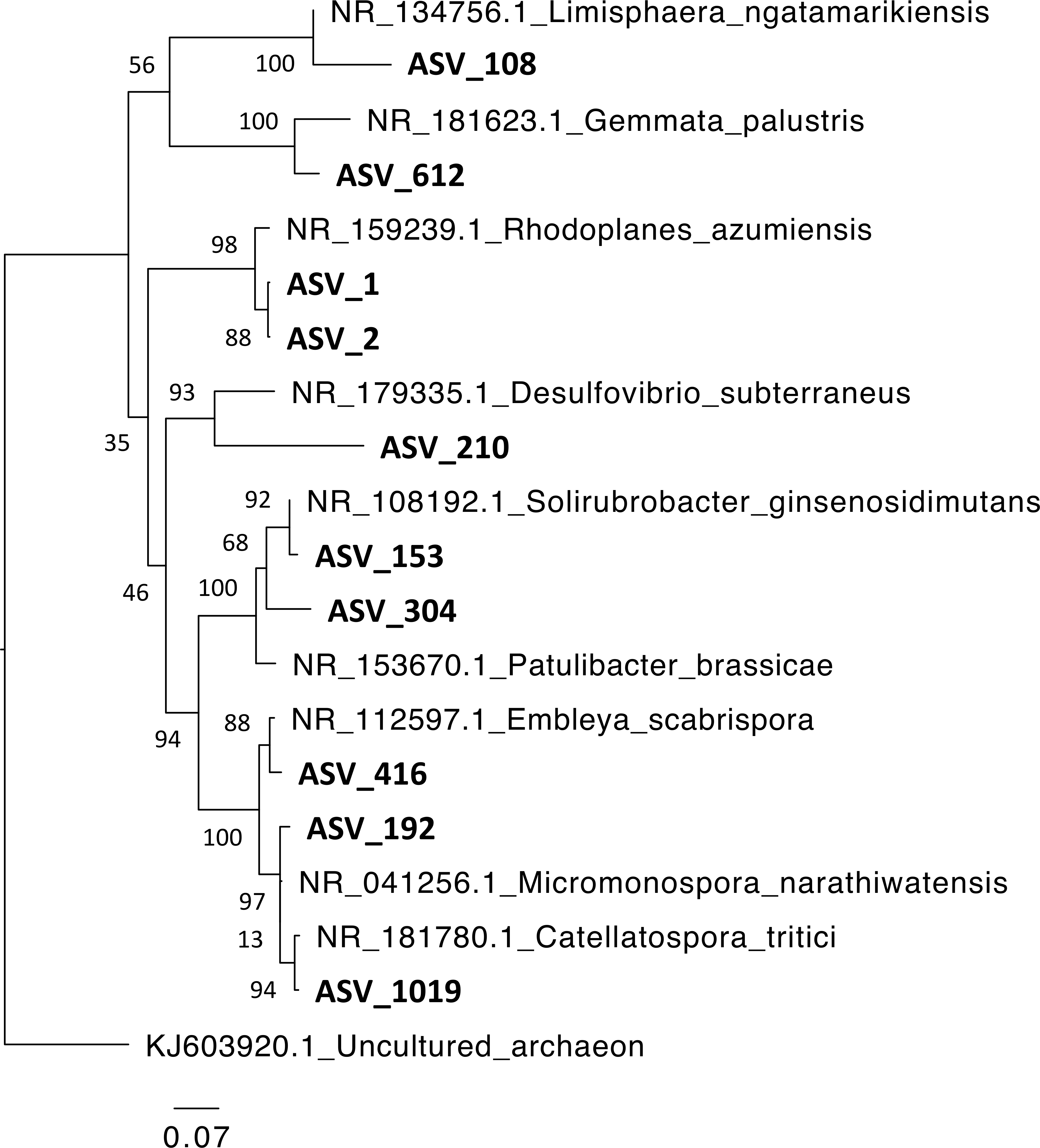

Supplement: Supplementary file 1 [file microorganisms-11-00832-s001.zip › Figure S3.tiff]

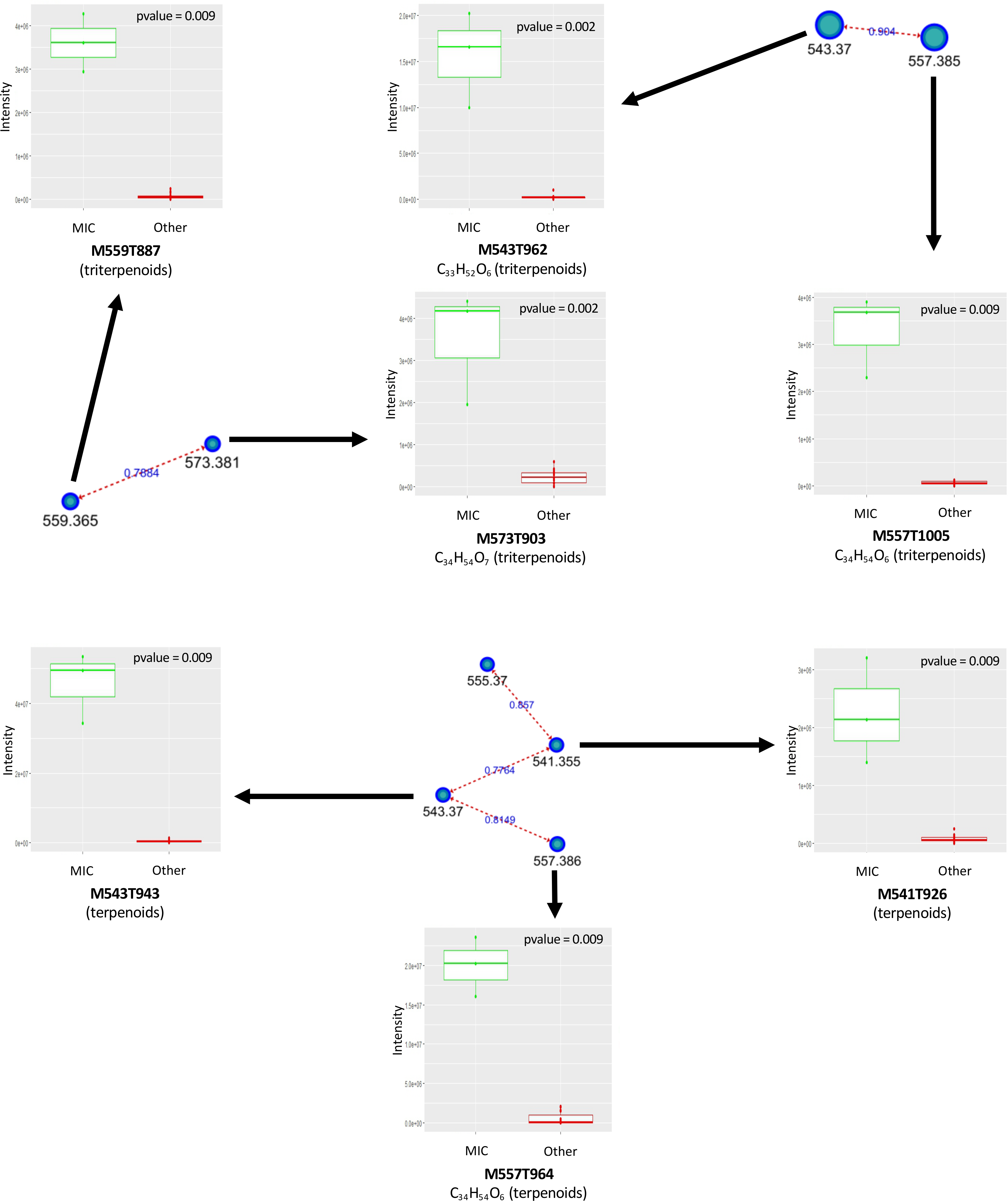

Supplement: Supplementary file 1 [file microorganisms-11-00832-s001.zip › Figure S4.tiff]
